# Impact of oral motor task training on corticomotor pathways and diadochokinetic rates in young healthy participants

**DOI:** 10.1111/joor.13349

**Published:** 2022-06-30

**Authors:** Noéli Boscato, Hidetoshi Hayakawa, Takashi Iida, Yuri M. Costa, Simple Futarmal Kothari, Mohit Kothari, Peter Svensson

**Affiliations:** ^1^ Department of Restorative Dentistry, School of Dentistry Federal University of Pelotas Pelotas Brazil; ^2^ Section for Orofacial Pain and Jaw Function, Department of Dentistry and Oral Health Aarhus University Aarhus Denmark; ^3^ Division of Oral Function and Rehabilitation, Department of Oral Health Science Nihon University School of Dentistry at Matsudo Matsudo Japan; ^4^ Department of Biosciences, Piracicaba Dental School University of Campinas Piracicaba Brazil; ^5^ Hammel Neurorehabilitation Centre and University Research Clinic, Department of Clinical Medicine Aarhus University Hammel Denmark; ^6^ Scandinavian Center for Orofacial Neurosciences (SCON) Aarhus Denmark; ^7^ JSS Dental College and Hospital JSS Academy of Higher Education and Research Mysore India; ^8^ Faculty of Odontology Malmö University Malmö Sweden

**Keywords:** diadochokinetic, language, motor learning, oral function, plasticity, transcranial magnetic stimulation

## Abstract

**Background:**

Studies addressing the training‐induced neuroplasticity and interrelationships of the lip, masseter, and tongue motor representations in the human motor cortex using single syllable repetition are lacking.

**Objective:**

This study investigated the impact of a repeated training in a novel PaTaKa diadochokinetic (DDK) orofacial motor task (OMT) on corticomotor control of the lips, masseter, and tongue muscles in young healthy participants.

**Methods:**

A total of 22 young healthy volunteers performed 3 consecutive days of training in an OMT. Transcranial magnetic stimulation was applied to elicit motor evoked potentials (MEPs) from the lip, masseter, tongue, and first dorsal interosseous (FDI, internal control) muscles. MEPs were assessed by stimulus–response curves and corticomotor mapping at baseline and after OMT. The DDK rate from PaTaKa single syllable repetition and numeric rating scale (NRS) scores were also obtained at baseline and immediately after each OMT. Repeated‐measures analysis of variance was used to detect differences at a significance level of 5%.

**Results:**

There was a significant effect of OMT and stimulus intensity on the lips, masseter, and tongue MEPs compared to baseline (*p* < .001), but not FDI MEPs (*p* > .05). OMT increased corticomotor topographic maps area (*p* < .001), and DDK rates (*p* < .01).

**Conclusion:**

Our findings suggest that 3 consecutive days of a repeated PaTaKa training in an OMT can induce neuroplastic changes in the corticomotor pathways of orofacial muscles, and it may be related to mechanisms underlying the improvement of orofacial fine motor skills due to short‐term training. The clinical utility should now be investigated.

## Graphical abstract

Impact of oral motor task trainig on corticomotor pathways and diadochokinetic rates in young healthy participants.

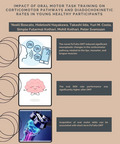

## 1 INTRODUCTION

Neuroplasticity and motor cortex integration play an important role not only in the generation and control network of oral functions (e. g., biting, chewing, swallowing, and speech^1^ but also in the improvement of motor skills.^2^, ^3^ Previous studies have suggested a repeated training in an orofacial motor task (OMT) can trigger neuroplastic changes in the corticomotor control of the tongue and jaw‐closing muscles and that the amplitude of the motor evoked potentials (MEP) can reflect the neuronal excitability in the muscle motor cortex in response to transcranial magnetic stimulation (TMS).^4^, ^5^, ^6^, ^7^

The oral diadochokinetic (DDK) rate, or the number of syllables spoken in a given period, has been frequently used to assess neuromotor abnormalities and orofacial motor control impairments that precede impairment in speech and masticatory functions.^8^, ^9^ Abnormal DDK rate performance might indicate central or peripheral nervous system disorders.^10^ Oral DDK rate is also suited to reveal an improvement of basic motor capabilities and language after specific OMT.^8^ This quantitative analysis must be done carefully by comparing the individual's speech task performance because different kinds of articulators, such as the upper and lower lips, jaw, and tongue musculatures are precisely coordinated in the speech production and oral function for different muscles and phonetic stimuli.^9^, ^10^, ^11^, ^12^, ^13^ Investigating this muscular cooperative mechanism is imperative not only for constructing speech models but also for assessing the neural activity involved in speech production and basic oral motor capabilities.^14^, ^15^, ^16^ The PaTaKaRa™ app is a modern, innovative, and reproducible method for checking oral frailty status and improving the oral function using a series of single syllables (i.e., Pa, Ta, and Ka) pronounced as rapidly as possible.^17^

Few studies have demonstrated changes in corticomotor pathways representing the lips,^18^, ^19^ tongue, and jaw muscles after OMT.^4^, ^5^, ^6^, ^7^, ^20^ However, no studies have addressed the modulation‐associated plasticity and lip, masseter, and tongue motor representations in the human motor cortex and oral DDK rate after Pa, Ta, and Ka single syllable repetition. Therefore, it would be important to assess the effect of a novel PaTaKa training in an OMT in healthy participants with normal speech and oral function performance to better understand the basic physiological mechanisms related to improvement of motor skills and potential neuroplastic changes. Subsequently, pathological speech and oral function performance can be evaluated in clinical populations.^8^, ^16^

Therefore, this study investigated the impact of a repeated training in a novel PaTaKa DDK OMT on corticomotor control of the lips, masseter, and tongue muscles in young healthy participants. The tested hypothesis was that 3 consecutive days of repeated PaTaKa OMT would be sufficient to increase the excitability of the corticomotor representation of the target muscles and the rate of oral DDK in healthy participants.

## 2 MATERIALS AND METHODS

### 2.1 Participants

Twenty‐two healthy individuals (63.6% women) with a mean age (SD) of 30 years (7.4) and with different first languages (e.g., Chinese, Brazilian, English, Spanish, Hungarian, French, Danish, Japanese) were recruited into this experimental study. The volunteers were recruited by advertising on a webpage of the Section for Orofacial Pain and Jaw Function (http://odont.au.dk/om‐odontologi/sektioner/kof/) with an invitation to participate in the study. Inclusion criteria were defined as age >18 years, good systemic health, and no ongoing orofacial pain or other types of chronic pain in the last 6 months.^21^ The exclusion criteria were the presence of dental or medical illness, regular intake of medication such as antidepressants, anticonvulsants, or nonsteroidal anti‐inflammatories, any diagnosis of psychiatric or personality disorders, and the presence of contraindications to TMS (presence of metal implants in the head, implanted electronic devices, pregnancy, and history of epilepsy).^22^ It was expected that a medium effect size of *f* 0.25 for the differences in corticomotor excitability would be sufficient for detecting significant effects, considering the within‐between interactions with a power of 80% and a significance level of 5%. Therefore, the sample size estimation was at least 16 participants. Prior to the experiment, all participants gave written informed consent. The study was conducted in accordance with the Helsinki Declaration II and was approved by the Human Local Ethics Committee (approval No.1‐10‐72‐417‐17).

### 2.2 Outcome variables

The following outcomes were assessed: (a) MEP amplitude and corticomotor mapping of the lips, masseter, tongue, and first dorsal interosseous (FDI, used as an internal control); (b) oral DDK rate (primary outcomes); and (c) self‐reported motivation, fun, pain, fatigue, and difficulty scores from numeric rating scale (NRS) (secondary outcomes).

### 2.3 Experimental procedures

**FIGURE 1:**
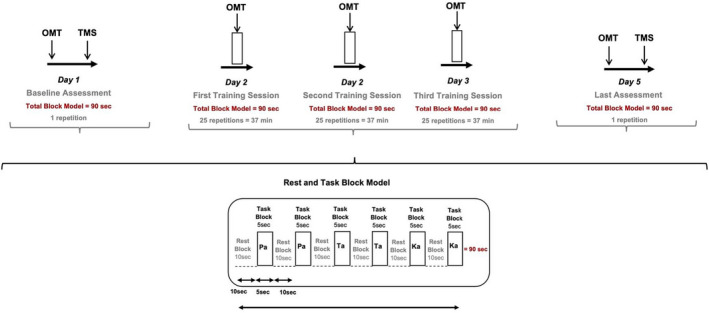
Study design: This study consisted of 5 sessions, 2 testing sessions of MEP recordings, and 3 training sessions as follows: Day 1, test session recording MEP at baseline, before the OMT; Days 2, 3, and 4, first, second, third training sessions; and Day 5, testing session recording MEP after the third training session. In the two testing sessions, at baseline and last session, the pre‐activation of the target muscles was performed with 1 repetition of the block model with a duration of 90 s before MEPs recording by TMS. In each training session, participants repeated 25 times the block model with a duration of 90 s, providing a total of 2250 s or 37 min of training. PaTaKa OMT, PaTaKa orofacial motor task; TMS, transcranial magnetic stimulation

This study consisted of 5 sessions, 2 testing sessions recordings lips, masseter, tongue, and FDI MEPs evoked by TMS, and 3 training sessions: Day 1, test session recording MEP at baseline, before OMT training; Day 2, 3, 4, first, second, and third training sessions; and Day 5, testing session recording MEP after the third training session. The PaTaKa training in an OMT followed the PaTaKaRa™ app exercises (Sunstar Suisse SA, Europe, 2020) developed for training orofacial muscles and checks how many times participants can repeat Pa, Ta, and Ka single syllables in rapid succession as follows: Participants alternately performed a 5‐s task block pronouncing a predefined single syllable sequence in the specific order Pa, Ta, and Ka, as rapidly as possible and a 10‐s rest block providing a block model with a duration of 90 s (i.e., 10‐s rest block performed 6 times alternated by 5‐s task block pronouncing Pa, Ta, and Ka 2 times each single syllable). In the 2 testing sessions (i.e., Days 1 and 5), before recording MEP evoked by TMS, a pre‐activation of the target muscles was performed with 1 repetition of the block model with a duration of 90 sec; while in the training sessions (i.e., Days 2, 3, and 4), participants repeated 25 times the block model with a duration of 90 sec, providing a total of 2250 s or 37 min of training, as shown in Figure 1.

The DDK rate and the self‐reported NRS scores were obtained from each participant at baseline and immediately after repeated training in an OMT.^23^ Oral DDK was defined as the maximal rapid syllable repetition rate per second to evaluate articulatory oral motor skills at the lips, masseter, and tongue musculature sites.^16^ According to the PaTaKaRa™ app, the DDK rate was classified as weak (up to 4.9), fine (5 to 7.9), or excellent (≥8). The self‐reported motivation, funny, pain, fatigue, and difficulty scores were rated on a 0–100 NRS, where 0 means “no” and 100 means “the highest motivation, fun, pain, fatigue, and difficulty scores”.^23^

Representative EMG recordings were obtained from a single participant illustrating details from lips, masseter, and tongue EMG activity during the execution of the PaTaKa OMT. The participant was instructed to start with a 5‐s rest block, and after, pronouncing each predefined Pa, Ta, and Ka single syllable with a 1‐s task block alternating by a 1‐s rest block with a duration of 10‐s, providing in total a 15‐s block model illustrating the voluntary activation of the masseter, tongue, and lip muscles from the intersyllable pauses and the syllable with peak intensity during the execution of the training task and pronouncing each predefined single syllable Pa, Ta and Ka. The zero‐amplitude indicates the repositioned threshold from the intersyllable pauses and the syllable with increased peak intensity. The points to the onset are marked above the zero‐amplitude line, and the points to the mid‐range activity below the line, as shown in Figure 6.

Motor evoked potentials recording by focal TMS was performed in the two testing sessions using a Magstim 200 stimulator (Magstim Co. Ltd.). The participants were seated on a dental chair in a supine position supported by a headrest. A flexible silicone cap was placed over the head in a standardised way based on anatomical markers and in accordance with the International 10–20 Electrode Placement System guidelines.^22^, ^24^, ^25^ EMG activities were recorded from the right side of the tongue dorsum, masseter, lip, and first dorsal interosseous (FDI) muscles.^6^, ^7^, ^26^ The electromyographic (EMG) activity prompted by the TMS was recorded from the masseter, lip, and FDI using bipolar surface electrodes (Neuroline 720, Ambu), while disposable self‐adhesive silver chloride electrodes (Alpine bioMedic Apa) were placed in the tongue. During MEP recordings, a ground electrode was placed around the left wrist connected to the amplifier to allow bipolar registration of contralateral MEPs. The EMG signals were amplified, filtered (10 Hz–3 kHz), and stored on Viking Select (Nicolet EDX, Natus Medical Inc.). TMS–MEP stimulus–response curves and motor maps were constructed using the peak‐to‐peak amplitude of the non‐rectified TMS–MEPs quantified in the time frame of 30 ms beginning 3 ms after the TMS artefact.^27^

TMS pulses were delivered with a 5‐cm diameter figure‐of‐eight stimulation coil to the left side of the scalp.^6^, ^26^ The stimulator coil was oriented 45° obliquely to the sagittal midline so that the induced current flowed perpendicular to the estimated alignment of the central sulcus.^5^, ^26^ The stimulator coil was moved over the left hemisphere with constant stimulus intensity to determine the optimal position to elicit maximal MEP peak‐to‐peak amplitudes in each target muscle. Three markings on the coil helped identify the position in relation to the scalp sites. The scalp site at which EMG responses were evoked in each target muscle at the lowest stimuli strength was determined and marked with a pen on the cap.^6^, ^7^

In accordance with previous studies, MEP in the FDI (used as an internal control) could be evoked by stimulation of the scalp about 1 cm anterior to the Cz line and about 6 cm lateral to the midsagittal plane.^6^, ^7^, ^20^ For MEPs recording from the masseter, surface electrodes were placed 10 mm apart and parallel to the main direction of the muscle fibres, along the central part of the masseter muscle, i.e., midway between the anterior and posterior borders (determined by manual palpation) and the origin (inferior to the bony margin of the zygomatic process) and insertion (superior and anterior to the mandibular angle and lateral surface of the ramus of the mandible).^27^, ^28^ The MEPs in the right masseter muscle could be evoked by TMS with the stimulation of discrete areas of the left scalp, approximately 4 cm anterior to the Cz and 9 cm lateral to the mid‐sagittal plane.^29^ To ensure standardisation of background activation across subjects, participants kept a special biting device between the anterior teeth calibrated to 10‐N force when the two parts were in contact. It provides a constant pre‐activation of the muscle, which is required for eliciting masseter MEPs, and also continuous feedback on the targeted force level allowing a constant and reproducible activation of the jaw‐closing muscles.^20^, ^28^ In order to locate the lip representation of the corticomotor area, one electrode is attached to the right corner of the upper lip and another to the right corner of the lower lip, with MEPs being recorded in the orbicularis oris, starting from a site 3 cm lateral and 1.5 cm anterior to the FDI representation, along a straight line towards the corner of the left eye.^18^, ^19^ For tongue MEP recording, electrodes were placed on the right side of the dorsal surface of the tongue (2–3 mm from the midline, 10 mm from the tongue tip) with an interelectrode distance of 2 cm.^6^ MEPs were evoked by stimulation of discrete areas of the left scalp, approximately 2–3 cm anterior to the Cz and 7–8 cm lateral to the midsagittal plane.^6^, ^26^

The resting motor threshold contralateral to stimulation was measured at all time‐points in the relaxed muscles (i.e., tongue, lip, and FDI) and active motor threshold (i.e., responses evoked during the tonic contraction of masseter) with the use of descending and ascending method of limits. It was defined as the minimum stimulus intensity that produced 5 out of 10 discrete MEPs for each target muscle with peak‐to‐peak amplitude (i.e., greater than or equal to 10 μV for the tongue, lip, and masseter and 50 μV for the FDI) considering background EMG activity clearly discernible on the monitor from 12 consecutive stimuli.^6^, ^18^, ^20^, ^26^ The peak‐to‐peak amplitudes of the MEPs (μV) were used to assess corticomotor stimulus–response (S–R) curves and map the motor cortex for sites from which the MEPs could be evoked. S–R curves were constructed in steps of motor threshold, from −10%, +20% to +60% (i.e., respectively at 90%, 100%, 120%, and 160% motor threshold), in a randomised order, where the motor threshold was the resting or active motor threshold measured at the specific time of creating the S–R curve. Given that comparisons are made on separate days from pre‐ to post‐training of the target muscles, if the motor threshold had changed after training, the post‐training motor threshold was used as a reference for creating the post‐training S–R curve. MEP amplitude was the average of 12 stimuli delivered at each stimulus level, with an interstimulus interval of 10–15 s. After S–R curves, to create the corticomotor mapping, eight TMS stimuli were delivered to each grid site over the scalp identified by the flexible silicone cap marked with the 1–1 cm^2^ grid in an anterior–posterior and lateral–medial coordinate system. The stimulator output was set at 20% (120%) above the motor threshold, and the grid was stimulated in a fixed pattern, beginning at the center of the hotspot and then moving anterior, then posterior, at increasing and decreasing latitudes (sites typically covered 5 cm from the vertex [Cz] and 5 cm anterior and posterior to the interaural line) corresponding to at least 25 grids.^5^, ^6^ The corticomotor map areas (cm^2^) with MEP amplitudes greater than 10 μV (masseter, lip, and tongue) and 50 μV (FDI) were determined on the 1 × 1 cm^2^ grid.^5^, ^6^, ^28^ All assessments and experimental procedures were performed at the Department of Dentistry and Oral Health.

### 2.4 Statistical analysis

The outcome variables are reported as means and standard error (SE) unless otherwise noted. Normal distribution was assessed with the Shapiro–Wilk test, and log10 transformations were applied for the continuous variables when the results were significant, considering an alpha level of 5% (*p* < .05). Repeated‐measures analysis of variance (RM ANOVA) was calculated to assess differences in the MEP amplitude (log10 transformed values) considering two within‐subject factors, time — 2 levels (first testing session at baseline and last test session after PaTaKa OMT) and intensity of stimulation — 4 levels (90%, 100%, 120% and 160% motor threshold). Differences in corticomotor mappings areas for lip, masseter, tongue MEPs amplitude at 120% motor threshold were calculated considering the assessment time —2 levels (first testing session at baseline and last test session after PaTaKa OMT). Post hoc analyses were performed using the Bonferroni test. The RM ANOVA was also used to assess differences in the oral‐DDK rate considering two within‐subject factors, assessment time —2 levels (first testing session at baseline and last test session after PaTaKa OMT); and two test sessions and three training sessions time — 5 levels (first testing session at baseline, 3 training sessions, and last test session after PaTaKa OMT). Between‐group factors, single syllable — 3 levels (Pa, Ta, and Ka) differences were also calculated in another ANOVA model. Predicted probabilities by multilevel modelling (procedure mixed of Stata 14.2; StataCorp) were employed to identify Pa, Ta, and Ka oral‐DDK rate pattern (weak, fine, and excellent), respectively, for the tongue, lips, and masseter MEP amplitude at 90%, 100%, 120%, and 160% motor threshold considering the time at baseline and after PaTaKa OMT, describing the DDK rate and MEP amplitude response by time. Finally, the RM ANOVA was applied to assess differences in the motivation, pain, fun, fatigue, and difficulty NRS scores considering within‐group factors, time — 5 levels (at baseline, TD1, TD2, TD3, and after PaTaKa OMT), and between‐group factor (motivation, funny, pain, fatigue, and difficulty NRS scores) differences.

## 3 RESULTS

There were no significant between‐group differences in sex distribution (Bonferroni: *p* > .05). Considering within‐group differences, the self‐reported funny, pain, fatigue, and difficulty presented significantly lower NRS scores (i.e., less funny, pain, fatigue, and difficulty) as the training days progressed (Bonferroni: *p* < .01), Table 1.

**TABLE 1:** Self‐reported NRS motivation, pain, funny, fatigue, and difficulty scores at the first test session, 3 training sessions, and last test session after PaTaKa training in an OMT

NRS scores	Baseline	Training 1	Training 2	Training 3	After OMT
Motivation	64.3 (5.3)	63.0 (4.5)	58.2 (6.0)	54.7 (6.4)	62.2 (6.1)
Funny	60.4 (5.6)*	49.7 (6.1)	42.6 (6.4)	42.0 (7.0)	46.1 (7.1)
Pain	0.7 (0.5)	1.4 (0.6)	0.6 (0.3)	0.3 (0.2)	0.05 (0.04)*
Fatigue	3.3 (1.2)	13.5 (3.3)	10.3 (2.6)	9.1 (2.5)	1.1 (0.7)*
Difficuty	21.3 (4.7)	23.2 (3.5)	21.4 (3.4)	18.1 (3.3)	10.3 (2.8)*

*Note:* Repeated‐measures analysis of variance (NRS scores and time) followed by post hoc Bonferroni test (mean and SE).

(*) In the same row indicate significant within‐group differences considering NRS scores (*p* < .01).

Abbreviations: NRS, numeric rating scale; OMT, Orofacial motor task; SE, standard error.

**FIGURE 2:**
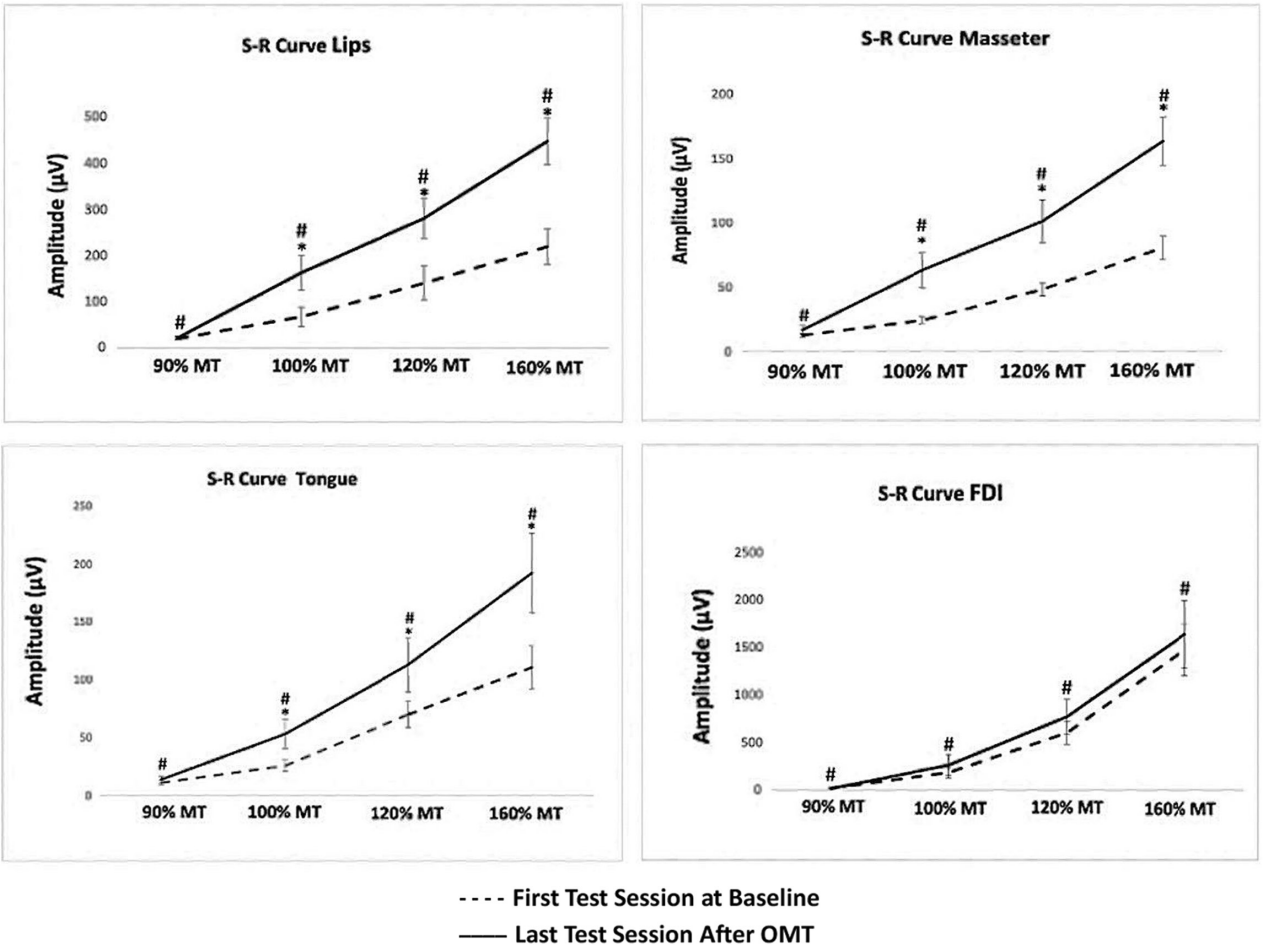
Stimulus–response (S–R) curves from lips, masseter, tongue, and FDI motor cortex (mean and SE) at baseline and after repeated PaTaKa training sessions in an OMT. Stimulus intensity is expressed in the percentage of MT. (*) indicates significant higher MEP amplitude differences in each intensity (i.e., within‐group), (*p* < .001). (#) indicates significant higher MEP amplitude between‐group differences considering 90%, 100%, 120% and 160% MT intensities, *(p* < .001). FDI, First dorsal interosseous; MEP, motor evoked potential; MT, motor threshold; PaTaKa OMT, PaTaKa orofacial motor task; SE, standard error; TMS, transcranial magnetic stimulation

**FIGURE 3:**
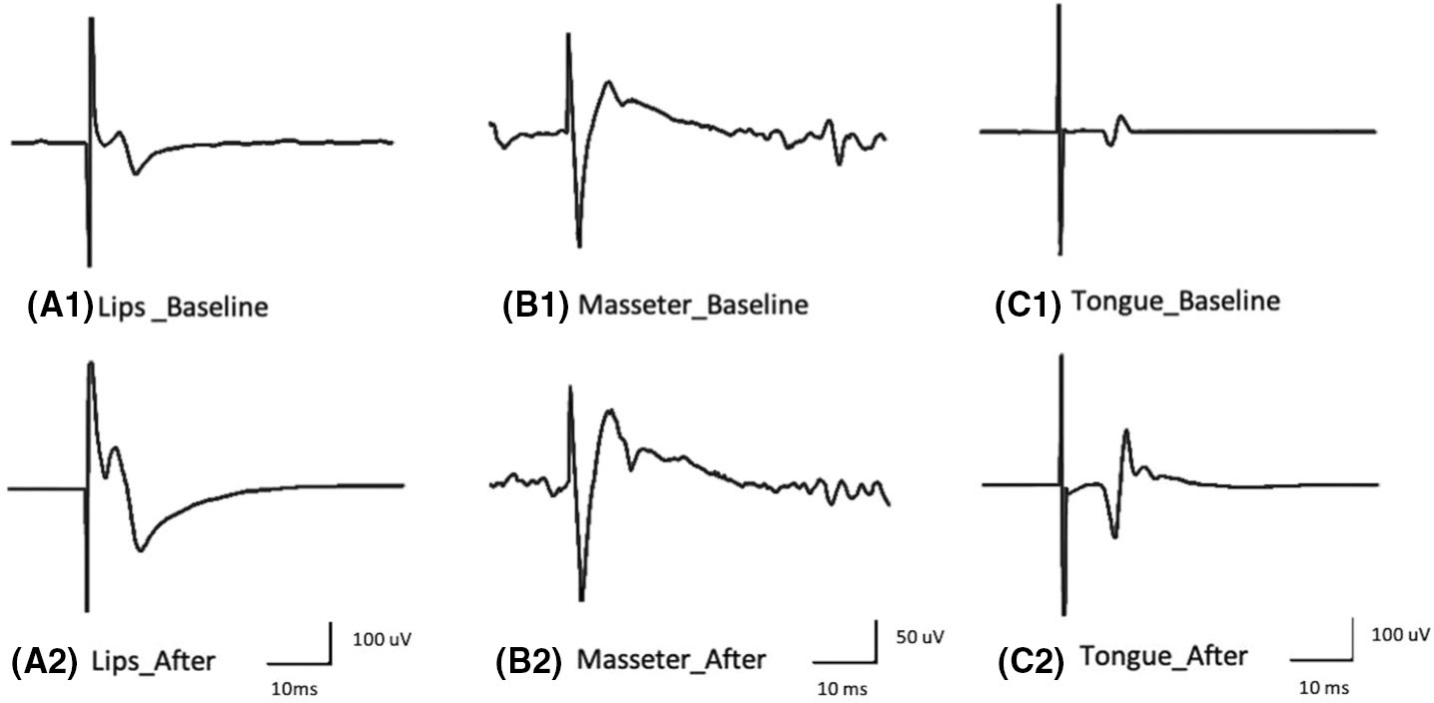
Example of average traces (12 sweeps) of MEP recording from lips (A1 and A2), masseter (B1 and B2), and tongue (C1 and C2) muscles from one participant, at baseline and after repeated PaTaKa training in an OMT, considering latency (ms) and amplitude (uV) at the stimulus level of 120% MT. MEP, motor evoked potential; MT, motor threshold; PaTaKa OMT, PaTaKa orofacial motor task; TMS, transcranial magnetic stimulation

**FIGURE 4:**
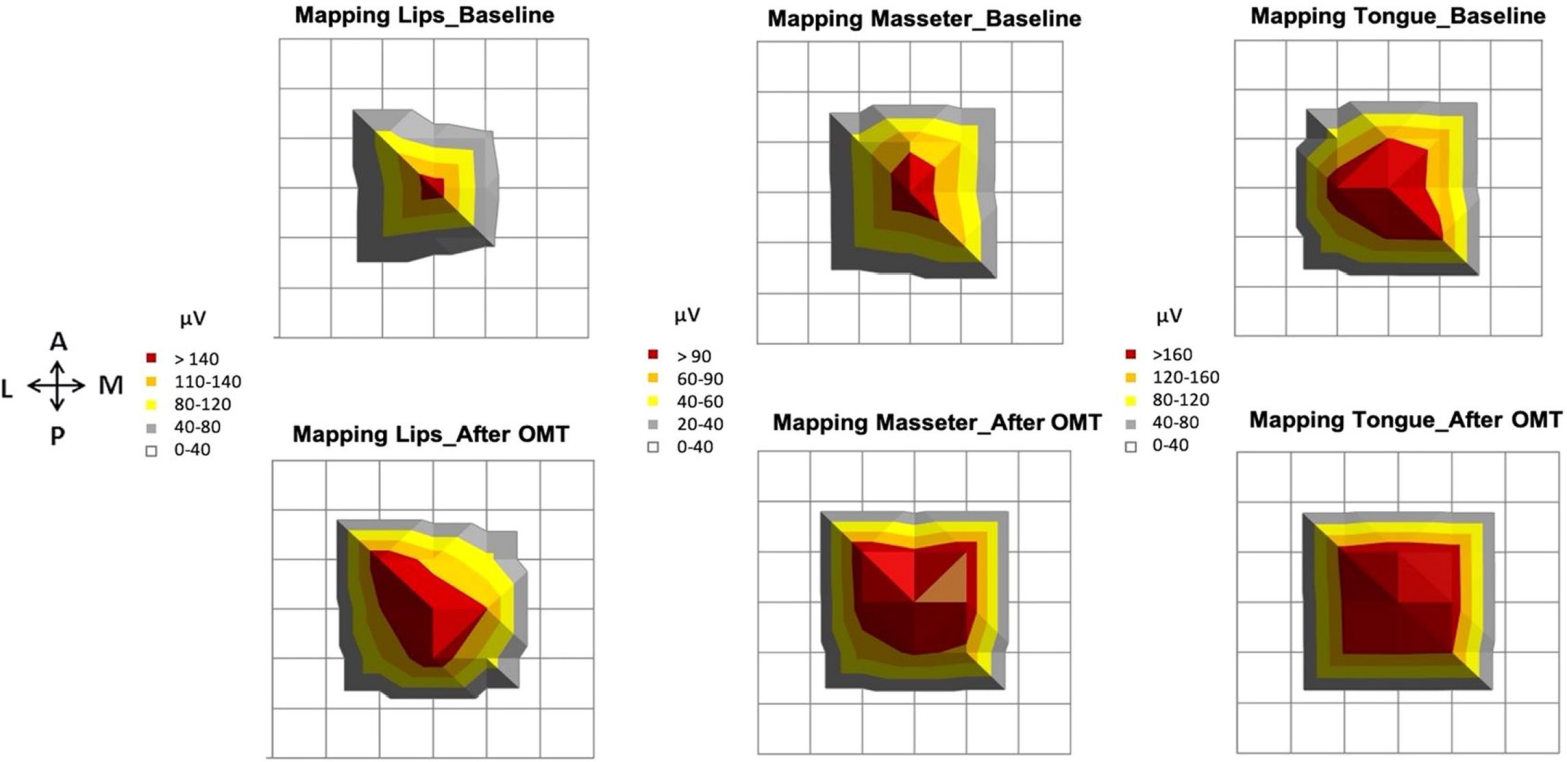
Illustrates the lips, masseter, and tongue corresponding corticomotor mapping areas (i.e., TMS of multiple scalp sites arranged in a one × 1 cm^2^ grid) at the first testing session and last test session after repeated PaTaKa training sessions in an OMT. Increased areas were displayed after OMT (Bonferroni: *p* < .001). Arrows indicate directions (A, anterior; P, posterior; M, medial; L, lateral). Zero on the *X*‐axis corresponds to the Cz line (interaural line). PaTaKa OMT, PaTaKa orofacial motor task; TMS, transcranial magnetic stimulation

Figure 2 shows the effects of the PaTaKa OMT on corticomotor excitability at 90%, 100%, 120%, and 160% motor thresholds. There was a significant effect of time and intensity on MEP amplitude, with higher values measured after PaTaKa training in an OMT at 100%, 120%, and 160% motor threshold when evaluated within‐group differences considering lips, tongue, and masseter muscles (Bonferroni: *p* < .001). Between‐group differences found the highest MEP values observed at the 160% motor threshold (Bonferroni: *p* < .001). Figure 3 shows average traces (12 sweeps) of one participant's lips, tongue, and masseter MEP amplitudes, with higher MEP values observed after repeated OMT than baseline. Figure 4 illustrates that participants revealed a significant increase in the corticomotor mapping areas ≥50% after OMT (Bonferroni: *p* < .001).

**FIGURE 5:**
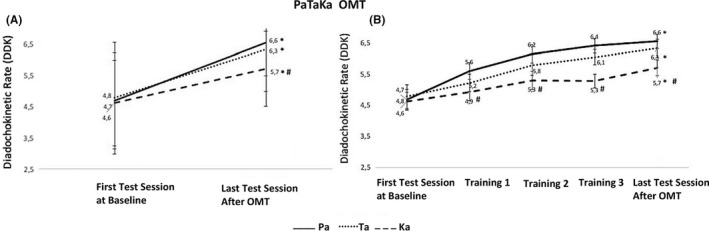
Show the oral‐DDK rate at the baseline and after repeated PaTaKa training sessions in an OMT (A); and oral‐DDK rate at baseline, each training session, and last test session after repeated OMT (B). (*) Indicates significant higher oral‐ DDK rate differences in each single syllable (i.e., within‐group), (*p* < .01). (#) Indicates significant lower oral‐DDK rate between‐group differences considering Pa, Ta, and Ka syllables, (*p* < .01). DDK, Diadochokinetic rate; PaTaKa OMT, PaTaKa orofacial motor task; TD, training session day

Significantly higher oral DDK rate within‐group differences after repeated OMT were observed considering each single syllable Pa, Ta, and Ka in the first test session, training sessions, and last test session (Bonferroni: *p* < .01), (Figure 5A,B). The syllable Ka presented a significantly lower DDK rate compared to Pa and Ta considering between‐group differences (Bonferroni: *p* < .01).

**FIGURE 6:**
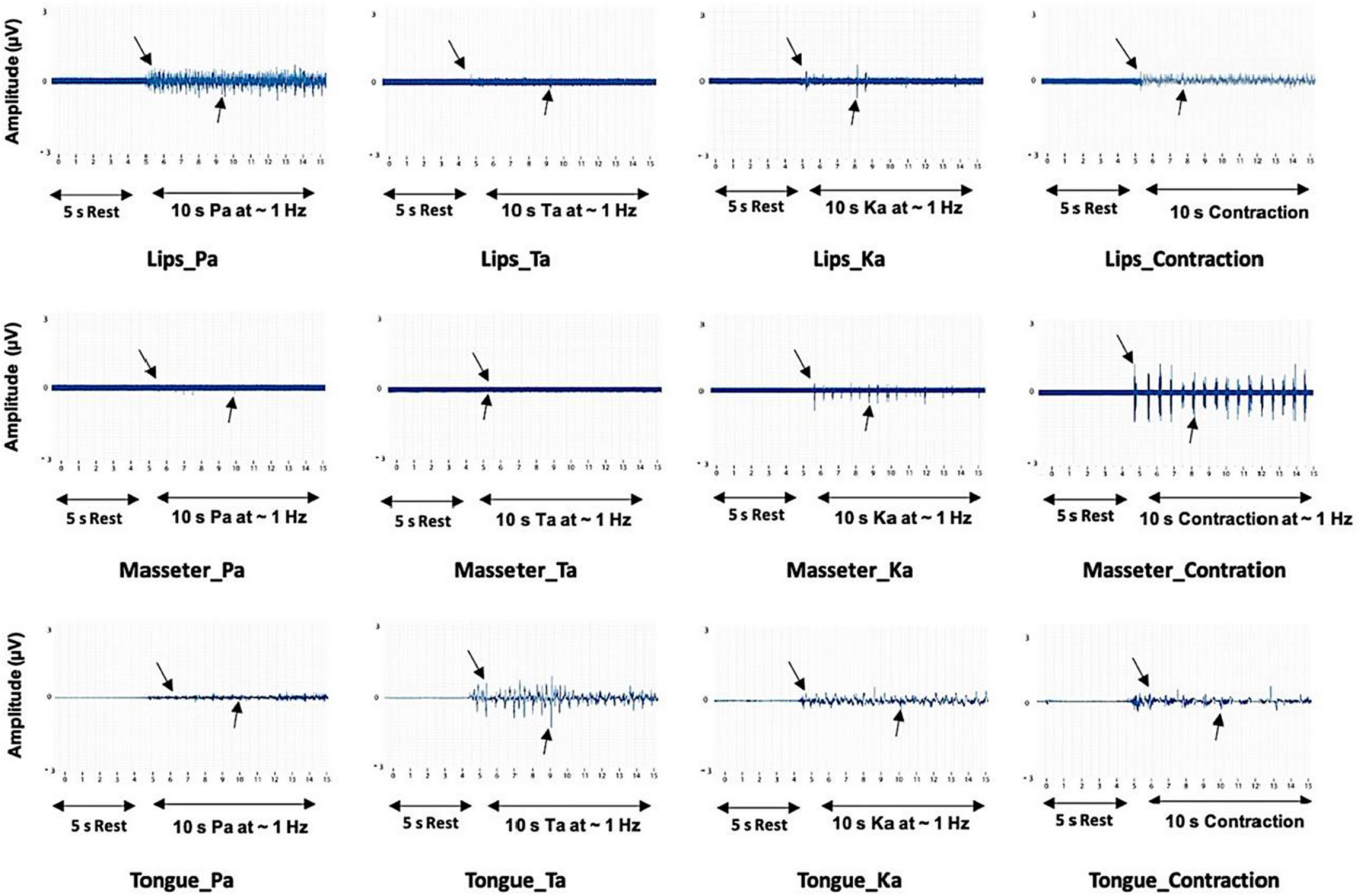
Overview of the execution of the repeated PaTaKa training in an OMT. Details of the lips, masseter (intramuscular recordings), and tongue electromyography (EMG) activity were recorded in a block model providing in total a 15 s of OMT as follows: the participant was instructed to start with a 5‐s rest block and after pronouncing each predefined Pa, Ta, and Ka single syllable with an intersyllabic interval of 1‐s of a rest block alternating by 1‐s of a task block, with a duration of 10‐s. The zero amplitude indicates the repositioned threshold from the intersyllable pauses and the syllable with increased peak intensity. The black arrow indicates the EMG activity. The first arrow (above the line) points to the onset, and the second arrow (below the line) points to the mid‐range activity. Contraction refers to a voluntary activation of the indicated muscle (as a control) either as a tonic contraction (lips and tongue) or as a repetitive dynamic contraction ~1 Hz (masseter). PaTaKa OMT, PaTaKa orofacial motor task

**FIGURE 7:**
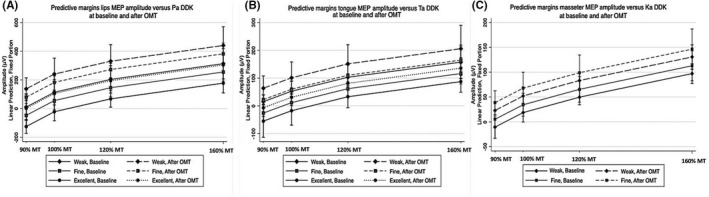
Predicted probabilities by multilevel analysis characterising lips (A), tongue (B), and masseter (C) MEP amplitude at 90%, 100%, 120%, and 160% MT and PaTaKa DDK rate (i.e., denominated ‘weak,’ ‘fine,’ and ‘excellent’) at baseline and after repeated PaTaKa training sessions in an OMT. MEP, motor evoked potential; MT, motor threshold; PaTaKa OMT, PaTaKa orofacial motor task

The EMG recordings illustrate intersyllable pauses and increased peak intensity from every single syllable repetition performed in the OMT. The images showed that the lips, tongue, and masseter had discrete higher activation when the syllables Pa, Ta, and Ka were pronounced, respectively (Figure 6). Multilevel analysis indicated that all participants, independent of their DDK rate baseline status (weak, fine, or excellent), experienced an increase in the MEP amplitude as the training days progressed (Figure 7A–C). This result indicates that the syllables repetition induces increased oral DDK rate in the target muscles and that DDK rate changes were associated with MEP amplitude changes at different stimulus levels. Characterising the lips, Figure 7A demonstrates that from Pa syllable pronunciation, participants with DDK rate denominated “weak” benefitted far more than those denominated “excellent” considering the baseline and after OMT. Similar findings were observed for the Ta DDK rate pattern characterising the tongue (Figure 7B). Regarding the masseter, the participants with “weak” and “fine” Ka DDK rates benefited from OMT. No participant experienced an “excellent” Ka DDK rate (Figure 7C).

## 4 DISCUSSION

This is the first study, to our knowledge, to show the linear relationship between training‐induced neuroplasticity, using a novel dynamic simulation of speech, and the establishment of a possible new neural pathway taken together with the lips, masseter, and tongue motor representations in the human motor cortex. Accordingly, the results of this study are important to understand whether and to what extent neuroplastic changes differ before (i.e., control session) and after a PaTaKa OMT in healthy participants with normal speech and oral function performance. Our findings confirmed the raised research hypothesis since significant differences were observed in terms of the training‐induced effect on MEP amplitude and DDK rate. These results produce novel insights into corticomotor neuroplasticity affecting lips, masseter, and tongue orofacial muscles during orofacial task performance.

In this study, the PaTaKa training using repeated single syllable repetitions significantly increased lips, masseter, and tongue MEP amplitude at 100%, 120%, and 160% motor threshold stimulus intensities (Figures 2 and 3) and corticomotor map areas (Figure 4). In terms of physiological mechanisms, these results indicate that the repetitive training in a novel OMT induces corticomotor neuroplasticity in healthy human participants. The significantly increased neuroplastic changes elicited by TMS and expressed as changes in TMS‐evoked motor potentials in the targeted orofacial muscles means that the repeated PaTaKa training produced neurophysiological effects in specific cortical areas. It was sufficient to modify neural activity patterns in the brain^30^ evoking higher MEP amplitudes picked up by EMG after the training sessions.^31^, ^32^ In contrast, the MEP amplitude and the motor cortex map related to FDI muscle (i.e., control hand muscle) did not significantly change, emphasising that FDI– MEP amplitude represents an internal control.^5^, ^26^ These results indicate that neuroplastic changes in the corticomotor pathway of the target muscles occur due to the training‐induced effect on cortical excitability. Significant differences between stimulus intensities were also revealed, with 160% motor threshold presenting the highest MEP values. These findings are consistent with previous data on stimulus–response functions of MEPs elicited by TMS in the masseter^4^ and tongue musculature from human studies.^5^, ^6^, ^26^ Animal studies also have showed neuroplasticity in the orofacial motor cortex due to OMT performance.^2^, ^33^ Interestingly, there were no significant sex differences related to training‐induced effects, suggesting that the PaTaKa training in an OMT effect may not differ according to sex.

The PaTaKa syllabic oral DDK rate performance increased significantly as the training days progressed, indicating an increase in the excitability of the corticomotor representation of the target muscles due to the short‐term training. This finding is in line with previous studies reporting a relative increase in the oral DDK rates following alternate‐word repetition in adults with normal speech using different programs.^10^, ^15^ Our results also showed oral DDK rate differences among a sequence of Pa, Ta, and Ka single syllable repetition, with the Ka syllable presenting significantly lower values than Pa and Ta (Figure 5A,B). These findings agree with a previous study using a similar dynamic simulation of speech in which a normal adult speaker provided a DDK rate ranging between 5 and 7 syllables per second, with a slower repetition rate of Ka than Ta or Pa.^8^ The vocalisation of the syllables Pa, Ta, and Ka activates and improves orofacial muscles as the training days progressed.^8^, ^17^ This result is also consistent with the hypothesis of lower control of oral articulatory movements for the Ka syllable since the EMG activity is not specifically linked to one specific muscle.^16^ The repetition of Ka is usually somewhat slower than Pa or Ta because posterior tongue movements are needed to make the Ka sounds.^8^, ^16^ It differs from Pa and Ta syllables pronunciation that directly involves the actions of the lips and tongue musculature.

In this study, oral DDK rates were corroborated by EMG activity, showing that the lips and tongue had the highest peak intensity related to Pa and Ta consonant‐vowel syllables pronunciation, as shown in Figure 6, while Ka had a more unspecific activity, including the masseter muscle. These findings suggest that the mechanical dynamic of lips, masseter, and tongue muscles play a significant and different role in the muscle activity coordinating multiple articulators during speech tasks^9^ and that the neuroplasticity induced by motor training may depend on the muscle group being trained and the specific task.^12^ In our study, the syllable Ka provided a more unspecific masseter muscle activity than Pa and Ta, respectively, in the lips and tongue muscles, as shown in Figure 6. This finding agrees with a previous study reporting that the masseter and temporalis jaw‐closing muscles are often active during speech, although the medial pterygoid muscle was the most active in all jaw‐closing movements.^32^

Data from a single participant illustrate EMG recordings with details from lips, masseter, and tongue activity demonstrated that the repeated PaTaKa training in an OMT provided stimuli that included intersyllable pauses during the rest block and increased peak intensity during the task block from every single syllable repetition, as reported in previous studies using similar motor speech programs.^16^, ^34^ In the process of collecting the data, the PaTaKaRa™ app demonstrated that it could be successfully applied to generate reproducible data and improve oral function in young healthy participants with normal speech function.^17^ Due to its intrinsic cyclic and relative simplicity, the app could have clinical and research utility to measure speech motor control for individuals with disordered speech.

The rationale to evaluate funny, fatigue, motivation, pan, and difficulty self‐reported NRS scores aims to analyse and critically summarise the participant's perception related to parameters being assessed as the training days progressed to guide future studies and prevent the repetition of issues negatively affecting the PaTaka training in an OMT. Based on the NRS scores, our findings revealed that the NRS funny scores significantly decreased as the training days progressed compared to baseline. Probably, participants perceived the beneficial effect of the training as making Pa, Ta, and Ka syllables pronunciation less funny. Less funny training paradigms may be helpful for OMT, keeping the participant more actively involved than funnier training.^5^ From the participant's perspective, the self‐reported NRS motivation scores indicated a continuous spectrum that positively influences the participant's motivation to participate in the study. Still, the NRS pain scores symptoms reported by the participants decreased across the study, with the lowest NRS pain scores observed in the third training and last sessions.

Interestingly, a decrease in the NRS difficulty scores was observed in the third and last session compared to baseline and first and second training sessions. This indicates that PaTaKa training in an OMT effectively improved muscle conditions as the training days progressed with a beneficial effect even in healthy participants. Finally, the highest NRS fatigue scores were observed during the training sessions of 37 min (first, second and third sessions) compared to 2 testing sessions with just muscle pre‐activation during 90 sec. While the NRS fatigue and difficulty scores decreased (Table 1), the OMT performance increased across the study (Figure 7). Therefore, the self‐reported decreases in the fatigue and difficulty scores matched the increases in OMT performance across the study.

Participants experienced an increase in the DDK rate after training (Figure 7A–C), with more differences found mainly for individuals exhibiting initially weak and fine DDK rates considering the temporal variation parameters at the baseline and after OMT. The participants with an excellent DDK rate at the baseline presented smaller DDK improvement since they already had an excellent DDK status pattern for a possible benefit from OMT. Those with weak and fine DDK rate patterns at the baseline had a higher DDK rate increase than those with an excellent pattern at baseline. In general, the Pa, Ta, and Ka repetition tasks may improve the motor speech system of individuals expressing minor or major dysfluencies and oral frailty. However, information on more specific functional implications of the PaTaKa OMT remains unknown from this study and will await further studies. Since the limited objective understanding of non‐pathological or normal motor speech skills and their disorders are available, the quantitative analysis of the oral function performance in young participants can assist in understanding and treating oral frailty.^15^, ^16^

In the present study, some limitations should be considered. First, this study assessed only PaTaKa training in an OMT in healthy participants. Individuals with poor cognitive and abnormal oral function were excluded since these factors could strongly affect the results.^34^ The authors understand that investigating the normal speech mechanism and recognising abnormal parameters early enough is imperative for constructing speech models and taking appropriate measures to prevent oral function decline and maintain our path to healthy aging; subsequently, the benefit from single syllable repetitions as trained in the current study in individuals with pathological speech should be evaluated in further studies addressing the relationship between cognitive and articulatory control from the PaTaKa training. Secondly, this study had a short follow‐up. The study design did not include testing sessions at 1 or more weeks after the last training session in order to establish how long the motor skill and neuroplasticity lasted. It would be important and provide new insights into the time‐course of corticomotor neuroplasticity, and related motor performance since the relationship between orofacial muscle improvement might be better determined in longer training paradigms and follow‐ups.^35^ However, a repetitive speech pattern might be less variable and easier to judge within a short period.^3^ Therefore, even though the information about the long‐term follow‐up after the last training session could have been interesting, it will await further studies. Indeed, further studies are needed to examine the effect of PaTaKa training in an OMT on motor cortex neuroplasticity to document the individual effects including participants of different ages (e.g., older people) and motor speech performance (e.g., abnormal motor speech). Since the elderly population is growing worldwide, it may help establish effective frailty prevention strategies to maintain their quality of life and reduce long‐term care needs. Therefore, attention should also be directed towards understanding the brain and neuroplastic changes as part of a comprehensive oral rehabilitation.^13^, ^36^

## 5 CONCLUSION

Our findings indicated that the novel PaTaKa OMT induced significant neuroplastic changes in the corticomotor pathway related to the lips, masseter, and tongue muscles. The oral DDK rate performance was also significantly higher after OMT. These findings suggest training‐induced plasticity in the corticomotor control of the target muscles and that it would most likely be related to mechanisms underlying the improvement of oral fine motor skills associated with short‐term training using single syllable repetition. The clinical utility should now be investigated.

## AUTHOR CONTRIBUTIONS

Peter Svensson and Mohit Kothari: Concept design and supervision. Hidetoshi Hayakawa and Noéli Boscato: Data acquisition. Noéli Boscato: Data analysis, prepared figures and wrote the manuscript. All authors, Peter Svensson, Hidetoshi Hayakawa, Mohit Kothari, Yuri M. Costa, Takashi Iida, Simple Futarmal Kothari, and Noéli Boscato contributed to the text of the manuscript, reviewed, edited, and approved the final submission.

## CONFLICT OF INTEREST

All authors declare that they have no known competing financial interests or personal relationships that could have influenced the work reported in this paper.

### PEER REVIEW

The peer review history for this article is available at https://publons.com/publon/10.1111/joor.13349.

## Data Availability

Data available on request due to privacy/ethical restrictions. The data that support the findings of this study are available on request from the corresponding author. The data are not publicly available due to privacy or ethical restrictions.
